# Personality Dimensions of Serbian Lifeguards

**DOI:** 10.3390/ijerph182412927

**Published:** 2021-12-08

**Authors:** Goran Dimitrić, Nebojša Maksimović, Elena Tabakova, Milorad Jakšić, Dejan Orlić, Selka Sadiković, Dea Karaba-Jakovljević, Nataša Zenić, Patrik Drid

**Affiliations:** 1 Faculty of Sport and Physical Education, University of Novi Sad, 21000 Novi Sad, Serbia; dimitrg@gmail.com (G.D.); nebojsam@uns.ac.rs (N.M.); lormida90@gmail.com (M.J.); dekiorlic@gmail.com (D.O.); 2 Institute of Sport and Physical Education, Russian State University of Physical Education, Sports, Youth and Tourism, 105122 Moscow, Russia; helena-avril@mail.ru; 3 Faculty of Philosophy, University of Novi Sad, 21000 Novi Sad, Serbia; selka.sadikovic@ff.uns.ac.rs; 4 Faculty of Medicine, University of Novi Sad, 21000 Novi Sad, Serbia; dea.karaba-jakovljevic@mf.uns.ac.rs; 5 Faculty of Kinesiology, University of Split, 21000 Split, Croatia; natasa@kifst.hr

**Keywords:** drowning, personality traits, neuroticism, conscientious, aggression

## Abstract

According to the World Health Organization’s (WHO) global drowning report (2017), drowning is the third leading cause of unintentional injury death worldwide. Drowning can occur anywhere there is water: oceans, seas, lakes, pools, bathtubs, rivers or water collection on the side of the road, etc. In many countries, there are drowning prevention programs for children and adults. The two most commonly used strategiesagainst drowning are the presence of lifeguards in public places and the use of protected areas that could prevent most of the drownings. The main aim of the present study is to examine the individual differences in a Big Five plus Two (BF+2) personality traits in lifeguards and non-lifeguards (including students). The subsample of lifeguards represented 122 male respondents who were, at the time of the survey, licensed as lifeguards (60.9%) or were in training for lifeguards—candidates (39.1%). The subsample of students represented 138 male respondents who were studying at the University of Novi Sad. The results indicate that lifeguards in comparison to students are more extraverted, open to experience, and conscientious, less neurotic, and aggressive. Both positive and negative valence are higher in student subsample. All of the above traits are desirable traits for people working as lifeguards.

## 1. Introduction

According to the World Health Organization’s (WHO) global drowning report [1], drowning is the third leading cause of unintentional injury death worldwide, accounting for 7% of all injury-related deaths. This report shows that age is one of the major risk factors for drowning, and the highest drowning rates are among children 1–4 years, globally. Males are especially at risk of drowning, with twice the overall mortality rate of females. Children who live near open water sources, such as ditches, ponds, irrigation channels, or pools, are especially at risk. Drowning accounts for 75% of deaths in flood disasters. Flood disasters are becoming more frequent, and this trend is expected to continue. Personnel under the influence of alcohol or drugs are also a risk. There are many more risk factors in drowning, such as lower socioeconomic status, being a member of an ethnic minority, lack of higher education, and rural populations, infants, left unsupervised or alone with another child around water, alcohol use, near or in the water, medical conditions—epilepsy, tourists unfamiliar with local water risks and features, etc. Death by drowning is the leading public health problem [2]. Drowning can occur anywhere there is water: oceans, seas, lakes, pools, bathtubs, rivers, water collection on the side of the road, etc. It is very important that people (especially children) are aware of the dangers that exist when staying in or around bodies of water. In many countries, there are drowning prevention programs for children and adults. Some methods of preventing child drowning involve swimming ability [3], caregiver supervision [4], lifeguard supervision on every pool [5], training parents to provide CPR [6], fencing private pools [7], educational programs [8], teaching school-age children swimming and water safety skills is a key intervention [1], etc. Moreover, increased swimming ability [9], the presence of lifeguards at every pool [5], creating a drowning chain of survival [10], adequate information, and signaling [11] are some of the preventive methods of drowning intended for adults. Drowning prevention measures should be multileveled, as only one preventive measure cannot be effective. In practice, it is possible to find several models for drowning prevention. One of them is water competency which could be a protective factor against drowning [12]. This relationship between swimming competency and drowning has never been scientifically proven [9]. Another model is the two strategies model. It consists of (a) proactive interventions, with the aim at reducing the risk of the environment, and (b) reactive preventive interventions with the aim at modifying the knowledge and behaviors of bathers, teaching them to detect dangerous situations and to improve their water skills [9,13]. The World Health Organization (WHO) has proposed some recommendations to prevent drownings, such as teaching school-age children basic swimming, water safety, and safe rescue skills [14]. The following five levels of prevention have been proven: four-sided pool fencing, life jackets, swim lessons, supervision, and lifeguards [15].

Among the many prevention strategies against drowning, the two most commonly used strategies, the presence of lifeguards in public places and the use of protected areas could prevent most of the drownings. Furthermore, drowning chances are significantly reduced in the presence of rescuers [16] and the presence of lifeguards at various types of beaches and swimming pools is most commonly noted as a preventive measure against drowning. In many countries, the presence of lifeguards is mandatory on all beaches and public pools. The presence of certified lifeguards is an important safety component where recreational swimming takes place [1,17].The role of lifeguards is multiple and of great importance. A lifeguard is defined as a person trained in lifeguarding, CPR and first aid skills, which ensures the safety of people at an aquatic facility by preventing and responding to emergencies [18]. A lifeguard is a specially trained and paid person who oversees safety, informs and educates visitors, enforces rules, uses special equipment and, if it is needed, rescues swimmers or non-swimmers in public swimming pools and bathing areas. Professional lifeguards should be mentally, physically, and emotionally prepared at all times to perform their job to standard. Characteristics of professional lifeguards include being knowledgeable and acquiring appropriate skills, reliable, mature, courteous and consistent, positive, professional, healthy, and physically fit [18]. Research conducted by [19] showed that the majority of the rescuers were 20–30 years old, strong, fit males with good vision and swimming ability. They renewed their lifeguard qualification, had work experience as a lifeguard, and they knew the dangers of the specific aquatic area. They worked in lifeguard teams rather than as solo lifesaver. Most of them were able to see the victim, recognize various signs of drowning and to react quickly despite the lack of reaction from bystanders. Lifeguards are working in difficult conditions. They are always under pressure when they respond to emergencies. In aquatic emergencies, where lifeguards work under pressure and make decisions in a short time could prevent lifelong damage and/or death. Quick decision-making and rescuing drowned people are the pressure that rescuers feel. Rescue and resuscitation of a drowning victim must occur within minutes to save lives and reduce morbidity in nonfatal drownings and underscores the critically time-sensitive role of the parent or supervising adult [15]. Lifeguards are exposed to potentially traumatic events as first responders. Job responsibilities all require some level of vigilance. Vigilance could act as a stressor in lifeguards due to their vigilant job responsibilities. Vigilance is a big problem for lifeguards. Boredom and monotony are present during the work of rescuers, and they are negative factors that affect the quality of work. Noise and lightning (inadequate or natural) could act as stressors which influence the quality of work and performance in lifeguards. Temperature also could be a kind of stressor. Lifeguards who work at an outdoor pool over the summer are at increased risk of heat stress due to exposure to direct sunlight. Lifeguards who work in an indoor recreational aquatics’ facility are exposed to temperatures which could present as a stressor due to the increase in humidity, poor ventilation, or lack of air conditioning [20]. The lifeguard’s quality of work is influenced by civility, incivility, and negative behavior and attitude (intimidating, tormenting, harassing, and anger). However, mutual relations have the greatest impact on the effects of work, and mutual trust is especially important. Having interpersonal relationships at work provides an understanding of a task, aims, and responsibilities for employees, which in turn has shown positive performance [21]. In practice, there are two types of lifeguarding. There is the more common pool and waterfront lifeguarding, where lifeguards work in locations without any kind of surf, such as pools, lakes, and ponds. Another type is beachfront lifeguarding, which allows the lifeguard to work on beaches facing the ocean. While rescues are never simple, in a pool or a lake, there are no waves, sea currents, or riptides. It is much easier to supervise people swimming in the pool than on the beachfront. These are general characteristics in which each rescue intervention differs.

The effects of their activities indirectly affect the economy by reducing the costs of different levels of medical care and society, reducing emotional trauma to the families of the drowned [16]. Lifeguards are expected to: monitor the aquatic environment and its visitors, prevent drowning, inform and educate visitors, enforce rules, use special equipment, provide first aid and CPR, etc. All of the above indicates that the duties of a lifeguard are extremely serious and require physical and especially mental abilities. Taking responsibility for other people’s lives and situations in which the rescuer can find himself (“fight” with the drowned during the intervention, potential death, etc.) requires mentally strong people.

### 1.1. The Personality of the Lifeguard

Limited research has been conducted into the personality structure and differences between lifeguards and non-lifeguards. It is recorded that lifeguards show similar personality qualities to other risky job professionals, such as firefighters, possibly due to job-related life and death situations [22]. For example, lifeguards are generally more drawn to risky behaviors and activities because of their thrill-seeking personality characteristics than non-lifeguards. Another personality dimension that is widely studied in the context of lifeguards and recruits is anxiety [22,23,24]. Furthermore, lifeguards, compared to non-lifeguards, are showing significantly lower cognitive, trait and state anxiety as well as lower somatic state anxiety [23,24]. Including some other personality dimensions, research showed that lifeguards, compared to other groups (general population, individuals engaged in physically risky sports and prosocial physically risky professionals), have the highest scores on Extraversion and Experience seeking and lowest on Neuroticism [22]. Lower aggression dimensions, on the other hand, are correlated with better communication skills [25] in lifeguards, which can be important in the selection process for lifeguards. Compared to other professionals (such as Navy cadets), lifeguards are demonstrating higher rigidity correlated with the over-role lower levels of stress [26].

### 1.2. Aims of the Present Study

The relative contribution of personality traits in shaping specific lifeguard’s behavior, as well as individual differences in various personality traits between lifeguards and other professionals, are yet to be understood. To the best of our knowledge, there are no previous studies considering personality dimensions beyond the Big Five personality framework.

Lexical research of personality, in general, is one of the leading paradigms in describing personality traits and captured the idea that language provides an opportunity to understand the structure of personality dimensions [27]. The Big Five model includes neuroticism, emotional instability, extraversion, sociability, gregariousness, enjoyment in social interactions, openness to experience, inquisitiveness and creativity, agreeableness, cooperativeness and trust and conscientiousness, order, self-discipline, and sense of duty [28]. Beyond the Big Five, some authors [29,30] proposed the seven-factor solution, with versions of the Big Five plus two factors describing Positive and Negative Valence. The Big Five plus Two was created in a series of taxonomic lexical studies in the Serbian language using the Tellegen-Waller procedure [31] and applying part of the Hofstee-Brokken procedure [27] to investigate the Serbian personality trait structure. The approach yielded a seven-factor personality structure, with five dimensions similar to Big Five dimensions, but with clear proof of two new evaluative factors: Negative Valence and Positive Valence factors [27]. Negative Valence includes indicators of Manipulative and Negative self-image and refers to the perception of oneself as an evil, frightful, and manipulative person, and Positive Valence includes indicators of Superiority and Positive self-image, referring to the self-perception as an exceptional and superior person [28,32]. Moreover, the Agreeableness dimension appeared as Aggressiveness in the Serbian lexical study, including markers of anger display, disagreeableness, and tough-mindedness. Two evaluative dimensions, Positive and Negative valence showed significant incremental validity in predicting various personality disorders [31,33] and personal attitudes [34], etc. It is an open question of how the perception of oneself differs between the general population and lifeguards as a helping professional.

Therefore, the main aim of the present study is to examine the individual differences in a Big Five plus Two (BF+2) personality traits in lifeguards and non-lifeguards (students).

In line with previous studies, we hypothesized that Extraversion would emerge as the highest personality trait in lifeguards, with lower Neuroticism and Aggression compared with the non-lifeguard group. Moreover, our theoretical expectation was that lifeguards might have higher Consciousness, according to a risky job that they are committed to. The relative contribution of Positive and Negative Valence is still to be examined.

## 2. Materials and Methods

### 2.1. Sample and Procedure

The subsample of students represents 138 male respondents who were studying at the University of Novi Sad. The age of the students ranged from 20 to 26 years (AS = 22.20; SD = 0.95). Data collection was conducted during 2020 at the University of Novi Sad. Participants were recruited by psychology students who administered the questionnaires for course credits in the Personality psychology course. Participation was voluntary. Respondents signed informed consent to participate in the research and were familiar with the objectives of the research. The questionnaires were distributed in paper-and-pencil format and took about 30 min to complete. The respondents were from the territory of the Republic of Serbia.

The subsample of lifeguards represents 122 male respondents who were, at the time of the survey, licensed as lifeguards (60.9%) or were in training for lifeguards—candidates (39.1%).The age of lifeguards and candidates ranged from 19 to 42 years (AS = 25.70; SD = 5.01). Data collection was conducted during 2020 at the Faculty of Sports and Physical Education, University of Novi Sad. Participants were recruited to engage voluntarily in the study by trained examiners on their regular training. They signed informed consent to participate in the research and were familiar with the objectives of the research. The questionnaires were distributed in paper-and-pencil format and took about 30 min to complete. The respondents are from the territory of the Republic of Serbia. Candidates and lifeguards were merged into 1 group of respondents due to the absence of differences between these 2 groups in the context of personality traits (λ = 0.949, F (7, 120) = 0.94, *p* > 0.05, η^2^p = 0.051), presence or absence of the interventions (λ = 0.921, F (7, 120) = 1.42, *p* > 0.05, η^2^p = 0.079) and places where they work or are in training (λ = 0.882, F (14, 238) = 1.11, *p* > 0.05, η^2^p = 0.061). Although they have different knowledge, skills, and experience in rescuing people, lifeguards and lifeguard candidates together represented a subsample of lifeguards. They are classified in the same group because they possess common qualities (traits) that enable them to engage in risky activities such as lifeguarding.

### 2.2. Instruments

Big Five Plus Two Questionnaire (BF+2) [31]. BF+2 was constructed on the basis of the second psycholexical study in the Serbian language, which was conducted using Telegen and Waller’s non-restrictive methodology for the selection of personality descriptions [35]. The questionnaire is intended to assess the seven dimensions of personality at the highest level of the hierarchy: five dimensions resemble Big Five, whereas Agreeableness is set in an opposite direction and includes indicators of anger manifestation, aggressive impulses, reactions, higher disagreeableness, and tough-mindedness. The additional two dimensions are evaluative dimensions—Positive and Negative Valence. Positive Valence includes positive self-concept and superiority, and Negative Valence includes negative self-concept and manipulative style and behavior. The questionnaire contains 184 items with a 5-point Likert-type answer scale: Neuroticism (*n* = 35, α = 0.93), Extraversion (*n* = 24, α = 0.82), Openness to Experience (*n* = 20, α = 0.78), Conscientiousness *n* = 28, α = 0.67), Aggressiveness (*n* = 30, α = 0.75), Positive valence (*n* = 25, α = 0.81) and Negative valence (*n* = 22, α = 0.78). The BF+2 questionnaire has also been applied and shown as a reliable measure in several previous studies which aimed to assess the basic dimensions of personality [28,36,37].

### 2.3. Data Analysis

The descriptive statistical method was applied to gain insight into the descriptive statistics indicators and parameters of the form (normality) of the distribution for the seven basic personality traits measured with the BF+2. Data visualization was graphically applied to show the differences between groups on the dimensions of the BF+2 questionnaire. Differences between the groups, in all analyzes, were examined using one-way multivariate analysis of variance (One-way MANOVA). The reliability of the VP+2 questionnaire dimensions was examined using Cronbach’s α coefficient [38]. All of the analyzes were performed in SPSS for Windows v22 [39].

## 3. Results

Descriptive statistical indicators for the whole sample, as well as for subsamples, are presented in Table 1. The values of skewness and kurtosis for the BF+2 questionnaire dimension range from conventionally acceptable values from −1.5 to 1.5 [40], both at the level of the whole sample, as well as at the subsample level. All of the analyses were performed on raw data.

Correlations between the dimensions of the BF+2 questionnaire, as well as the reliability of the dimensions themselves, are presented in Table 2. The reliability of the dimensions of the BF+2 questionnaire ranges from the good to the very good on the whole sample and the subsamples. The highest positive correlationon the whole sample is present between the Aggression and Negative valence dimensions, which is unsurprising because more aggressive people are usually more inclined to manipulate in order to achieve their own goal. The highest negative correlation is between Conscientiousness and Negative Valence. Low Conscience, which implies poor organization of activities, lack of inhibition in behavior and actions, etc., are associated with resorting to manipulative strategies to achieve a personal goal and a negative image of oneself. The correlations between the remaining dimensions are most significant and of moderate intensity. The pattern of correlations is very similar in the subsample of students and lifeguards, with the fact that in the subsample of lifeguards Positive Valence and Neuroticism do not have a significant correlation. In contrast, in the subsample of students, Aggression and Extraversion do not show significant correlations.

**Figure 1:**
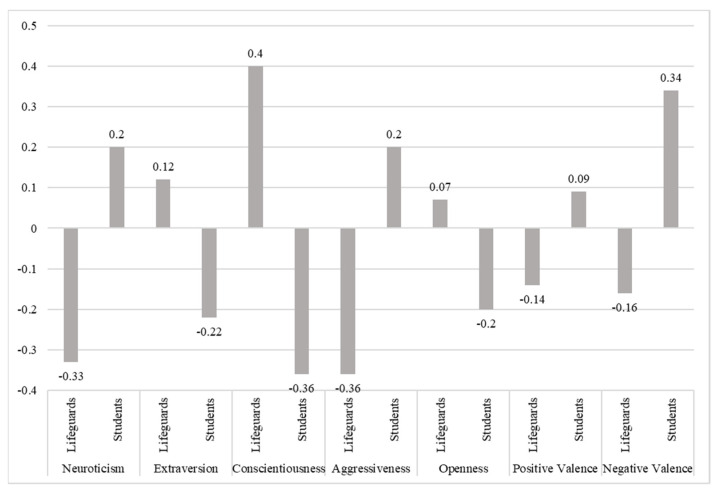
Differences between lifeguards and students on the dimensions of the BF+2 questionnaire (Z—scores).

The one-way analysis of variance (One-way MANOVA) was applied to examine the differences in personality dimensions between the student and the lifeguards. The independent variable was population (lifeguards and students), while the dependent variables were seven basic personality dimensions of the BF+2 questionnaire. The multivariate effect of the independent variable on the dependent variables was statistically significant (λ = 0.833, F (7, 376) = 10.76, *p* < 0.001, η^2^p = 0.167). Univariate effects and differences between groups are presented in Table 3 and Figure 1. Lifeguards achieve higher scores than students when it comes to the dimensions of Extraversion, Conscientiousness, and Openness to Experience, while students achieve higher scores than lifeguards in the dimensions of Neuroticism, Aggressiveness, Positive and Negative Valence. The effect sizes (partial eta-squared—η^2^p) are from 0.016 for Positive Valence to 0.084 for Aggressiveness.

## 4. Discussion

The analysis of the results of this study, which compares the personality traits of lifeguards and students, shows that lifeguards are more likely to be: extroverts, conscientious, and open to experiences, compared to students who are characterized by greater neuroticism, aggressiveness, positive and negative valence.

More pronounced values of extraversion, conscientiousness, and openness to experience in lifeguards compared to the students included in this research, are desirable traits. The lifeguard’s duty is to take care of other people while enjoying themselves by the water or in the water [18]. Extroverts care about other people [41], and that is a trait of lifeguards that this study confirms. The highest values of extroversion in lifeguards compared to other individuals engaged in physically risky sports and prosocial physically risky professionals were found [22]. Lifeguard conscientiousness is extremely important. A rescuer who wants to do his job well, efficiently, and safely for himself and others must be conscientious, responsible, careful. Any other approach to this responsible duty can be fatal. Openness to the experience of being a lifeguard indicates an individual’s willingness to face the risks and challenges that may occur.

Higher scores of neuroticisms in students included in this study indicate that they may react incorrectly in a stressful situation and potentially endanger themselves or another person. Decreased values of neuroticism in lifeguards indicate that they are more mentally ready to react correctly in stressful situations and able to make the right decisions. Pronounced aggression among students indicates that they are prone to aggressive reactions, which would be even stronger in stressful situations, such as rescuing drowned people. Aggression contributes to making quick and perhaps inaccurate decisions and reactions [42], which can be dangerous. Even verbal aggression in stressful situations is not a desirable trait. Lower values of lifeguards’ aggression indicate their knowledge and skill to approach the drowned person in order to calm them down and help them. Calmness and good negotiation skillsas well as patiently “building” a position when you need to “jump” on a panicked drowned person and prevent them from endangering the safety of the lifeguard and himself is one of the main characteristics of a good lifeguard.

Negative Valence implies that a person is prone to manipulation and has a negative opinion of himself. This trait is more pronounced in students than in lifeguards. Manipulation can contribute to improvisations, reactions that deviate from standard rescue procedures, and thus can reduce the effects of rescue. Lifeguards should have a positive attitude towards themselves and faith in themselves and their abilities to adhere to existing procedures so that they can adequately provide assistance in critical situations, but they must not neglect personal safety.

Positive Valence is higher in students, and they have a more pronounced sense of superiority and have a very nice picture of themselves. Lower values of lifeguards’ positive valence is a quality that indicates that they are aware of the risks of the activities they are engaged in, that they are not superior, and that their lives may be endangered.

The obtained results confirm the hypothesis that Extraversion is the highest personality trait in lifeguards, with lower Neuroticism and Aggressiveness compared with the students group.

## 5. Conclusions

The efficiency of rescuers’ work is influenced by working conditions, physical abilities, and psychological characteristics of rescuers. The aim of the present study is to examine the individual differences in a Big Five plus Two (BF+2) personality traits in lifeguards and non-lifeguards (students). Results of this study showed that lifeguards are more: extroverts, conscientious, and opened to experiences with lower neuroticism and aggression, compared with the non-lifeguard group, which confirms the hypothesis. Students were characterized by greater neuroticism, aggressiveness, positive and negative valence. The effects of such research can be recognized in a better organization of safety at swimming pools. In lifeguard courses, in addition to assessments of physical abilities, assessments of psychological characteristics also can be performed. Such assessments can select the best candidates with good physical abilities and appropriate psychological profile needed to perform such a responsible job, such as rescue.

## Figures and Tables

**Table 1:** Descriptive statistical parameters.

Population	Dimension	N	Min	Max	M	SD	Sk	Ku
Lifeguards	Neuroticism	122	35	125	72.42	20.29	0.494	−0.201
	Extraversion	122	69	119	98.92	10.34	−0.750	0.748
	Conscientiousness	122	76	138	111.49	13.06	−0.378	−0.022
	Aggressiveness	122	44	140	73.88	15.09	0.718	2.569
	Openness	122	51	94	77.09	9.40	−0.443	−0.130
	Positive Valence	122	45	111	84.78	13.96	−0.243	−0.336
	Negative Valence	122	24	71	38.40	10.11	1.140	1.164
Students	Neuroticism	138	42	132	83.28	19.72	−0.138	−0.355
	Extraversion	138	68	118	95.18	10.27	−0.225	−0.277
	Conscientiousness	138	69	137	100.63	13.09	0.061	−0.141
	Aggressiveness	138	52	128	82.61	13.80	0.122	0.183
	Openness	138	40	95	74.43	9.27	−0.352	0.737
	Positive Valence	138	54	123	88.04	11.82	0.093	0.956
	Negative Valence	138	23	87	43.87	11.31	0.814	0.885
Whole sample	Neuroticism	260	35	132	78.19	20.68	0.256	−0.442
	Extraversion	260	68	119	96.93	10.45	−0.444	−0.013
	Conscientiousness	260	69	138	105.73	14.14	−0.108	−0.381
	Aggressiveness	260	44	140	78.52	15.04	0.311	0.881
	Openness	260	40	95	75.68	9.41	−0.376	0.251
	Positive Valence	260	45	123	86.51	12.95	−0.165	0.291
	Negative Valence	260	23	87	40.30	11.09	0.930	0.866

Note: N—number of participants. Min—minimum value. Max—maximum value. M—arithmetic mean. SD—standard deviation. Sk—skewness. Ku—kurtosis.

**Table 2:** Correlations and reliability of the dimensions of the BF+2 questionnaire.

Population	Dimension	N	E	C	A	O	PV	NV
Lifeguards	Neuroticism	0.925						
	Extraversion	−0.322 **	0.806					
	Conscientiousness	−0.524 **	0.589 **	0.700				
	Aggressiveness	0.459 **	−0.213 *	−0.497 **	0.770			
	Openness	−0.244 **	0.717 **	0.501 **	−0.031	0.769		
	Positive valence	−0.040	0.395 **	0.225 *	0.234 **	0.522 **	0.780	
	Negative valence	0.692 **	−0.471 **	−0.527 **	0.558 **	−0.252 **	−0.006	0.758
Students	Neuroticism	0.921						
	Extraversion	−0.418 **	0.818					
	Conscientiousness	−0.292 **	0.410 **	0.667				
	Aggressiveness	0.374 **	−0.144	−0.037	0.697			
	Openness	−0.174 *	0.451 **	0.421 **	−0.060	0.788		
	Positive valence	−0.186 *	0.527 **	0.479 **	0.235 **	0.516 **	0.835	
	Negative valence	0.556 **	−0.311 **	−0.227 **	0.484 **	−0.154	0.007	0.768
Whole sample	Neuroticism	0.927						
	Extraversion	−0.400 **	0.816					
	Conscientiousness	−0.459 **	0.517 **	0.675				
	Aggressiveness	0.461 **	−0.220 **	−0.343 **	0.748			
	Openness	−0.235 **	0.588 **	0.474 **	−0.027	0.781		
	Positive valence	−0.072	0.425 **	0.271 **	0.259 **	0.491 **	0.810	
	Negative valence	0.640 **	−0.407 **	−0.416 **	0.550 **	−0.224 **	0.032	0.777

Note: N—Neuroticism. E—Extraversion. C—Conscientiousness. A—Aggressiveness. O—Openness. PV—Positive Valence. NV—Negative Valence. * *p* < 0.05; ** *p* < 0.01.

**Table 3:** Univariate effects and differences between groups.

Dimension	*F*-Test	Df	*P*	η^2^p	AM_STU_	AM_LG_	AM_DIF_
Neuroticism **	19.117	1.38	0.000	0.069	72.42	83.28	−10.86
Extraversion **	8.54	1.38	0.004	0.032	98.92	95.18	3.74
Conscientiousness **	44.55	1.38	0.000	0.147	111.49	100.63	10.86
Aggressiveness **	23.72	1.38	0.000	0.084	73.88	82.61	−8.73
Openness *	5.26	1.38	0.023	0.020	77.09	74.43	2.66
Positive Valence *	4.14	1.38	0.043	0.016	84.78	88.04	−3.26
Negative Valence **	16.74	1.38	0.000	0.061	38.40	43.87	−5.47

Note: Df—number of degrees of freedom. *P*—*p*-value. η^2^p—the magnitude of the effect expressed by the partial eta square. AM_STU_—arithmetic mean of a group that includes students. AM_LG_—arithmetic mean of a group that includes lifeguards. AM_DIF_—the difference between the arithmetic means of a group of students andlifeguards. * *p* < 0.05. ** *p* < 0.01.

## Data Availability

The data presented in this study are available on request from the corresponding author.
